# Terry Erwin’s legacy: from taxonomy and natural history to biodiversity research and conservation biology

**DOI:** 10.3897/zookeys.1044.68650

**Published:** 2021-06-16

**Authors:** Thorsten Assmann

**Affiliations:** 1 Institute of Ecology, Leuphana University Lüneburg, Universitätsstraße 1, 21335, Lüneburg, Germany Leuphana University Lüneburg Germany

## Abstract

No abstract

I first met Terry Erwin, already an icon of biodiversity research, at the fantastic 20^th^ International Congress of Entomology in Florence in 1996. His high level of scientific expertise, open mind, cooperative attitude, and enthusiasm for carabids overawed me immediately. Terry also radiated interest in other insects and whole ecosystems. Over the years, these traits have inspired many others, especially young scientists and students, as is clearly evident in the contributions of this issue (e.g. Spence 2021; Grammer 2021) and Kavanaugh (2020). In addition to Terry’s human qualities, his basic natural history approach to scientific research has significantly shaped his life’s work.

From my time as a graduate student, I literally devoured Terry’s publications, as his research made a deep impression on me. This was largely because Terry’s work was broad, ranging from classical taxonomy and natural history to sophisticated analyses of biodiversity and ecosystem services. I believe that Terry’s body of research is up-to-date and in many ways timeless, and that it will leave a lasting mark because of its broad organismic approach to biology. In this essay, I will briefly highlight what I regard as his most important research in a way that I hope will encourage others to read or even re-read it. That might be the way Terry would have been most happy to be remembered.

## Taxonomy

Terry Erwin started his research about carabid beetles with a taxonomic revision of the North American brachinines, a charismatic group of ground beetles with a famous explosive defense mechanism. Terry’s taxonomic revision with the description of new taxa appeared in 1970, but also included earlier works (Erwin 1965, 1969a, b, 1970a). His taxonomic contributions are characterized by especially comprehensive treatments of morphology, species descriptions, but also phylogenetic analyses following the principles laid out by Hennig (1969), which were a principal innovation in taxonomy at the time of Terry’s earliest studies. This approach underscores the influence of George E. Ball, Carl H. Lindroth, and P.J. Darlington, themselves giants in carabid systematics, who were his mentors. For carabidologists outside North America, it is especially significant that the relationships and systematics of related genera and higher taxonomic entities of the subfamily were included in Terry’s taxonomic revisions. This resulted in a work of lasting value that is still relevant today, after half a century, and used, not only as a basis for identifications, but for further systematic work.

Such treatments of taxonomically challenging ground beetle groups were a hallmark of Erwin’s scientific career. He worked both alone and with co-authors in this thorough way mainly on carabid taxa from the Americas. Work on the taxa *Leistus*, *Xystosomus*, and several *Agra* species groups provide excellent examples of the approach (e.g., Erwin 1970b, 1973, 1978, 1982a, b, 1983, 1984, 2002). He also worked on ground beetles on a world scale (e.g., Hiletini et al. 1985). These excellent taxonomic contributions undoubtedly represent the core of his scientific activity. Altogether, he has described 30 (sub-) genera and 438 species (ZooKeys Editorial Office 2015; Penev et al. 2021). Through his alpha-taxonomic achievements as well as the clarification of phylogenetic relationships, he contributed significantly to the systematics of the Carabidae and laid firm foundations for many future research activities.

**Figure 1.**
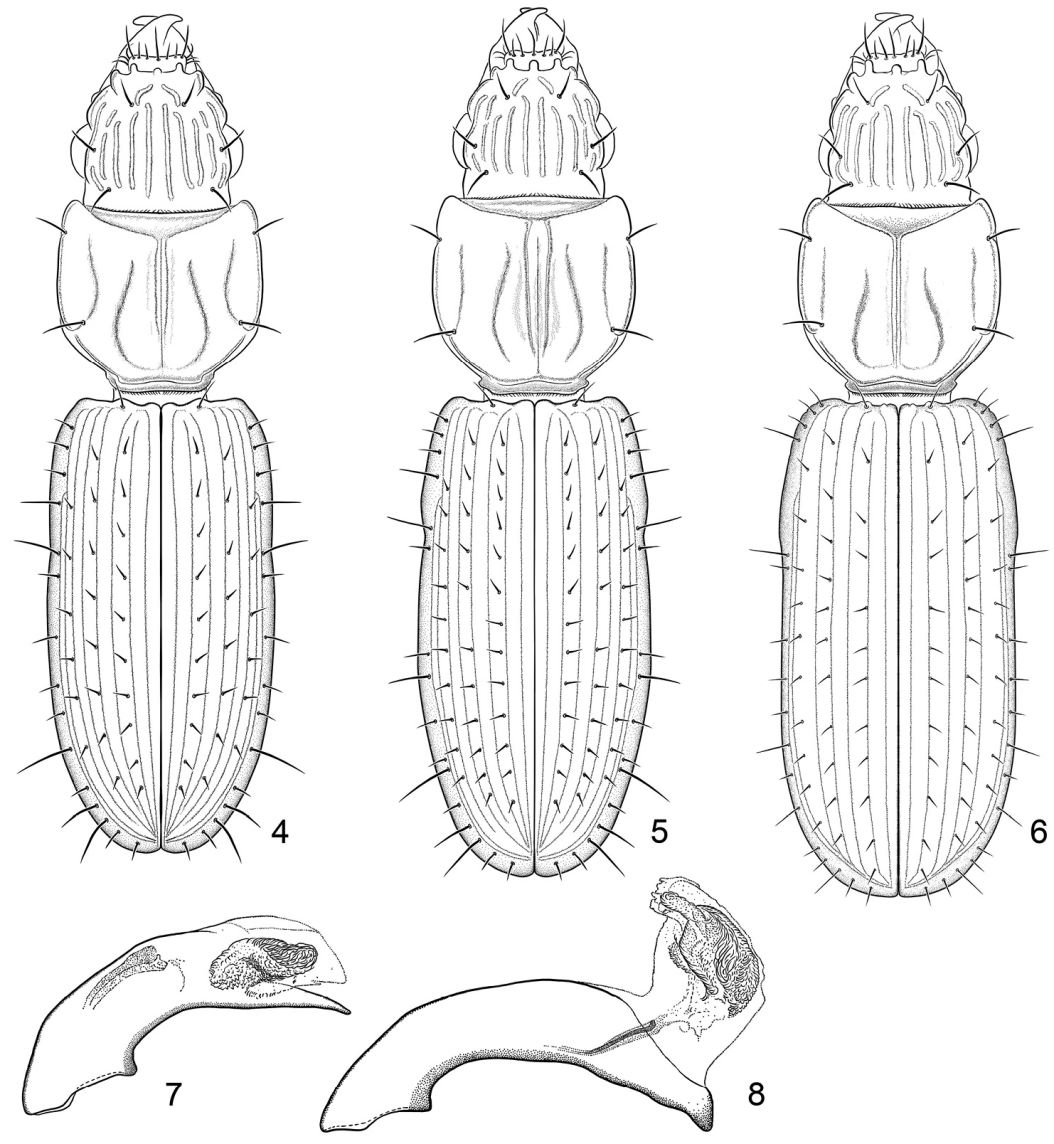
Example of Terry Erwin’s line drawings: dorsal aspects of three *Halocoryza* species and the median lobe of aedeagus of two *Halocoryza* species. From Erwin (2011: figs 4–8).

**Figure 2.**
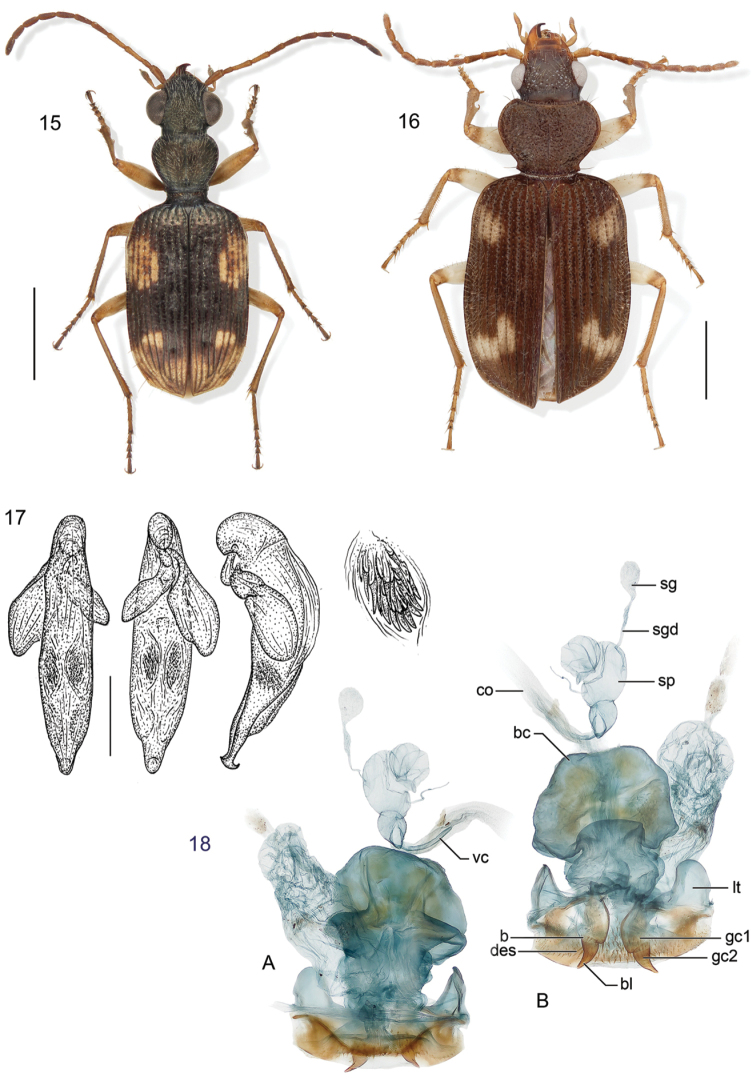
Example of Terry Erwin’s diverse illustrations: habitus, male genitalia (median lobe of aedeagus, with parameres) in dorsal, ventral, and lateral view with enlarged details of the internal sac and female genitalia from *Lachnophorus* species. From Erwin and Zamorano (2014: figs 15–18).

**Figure 3.**
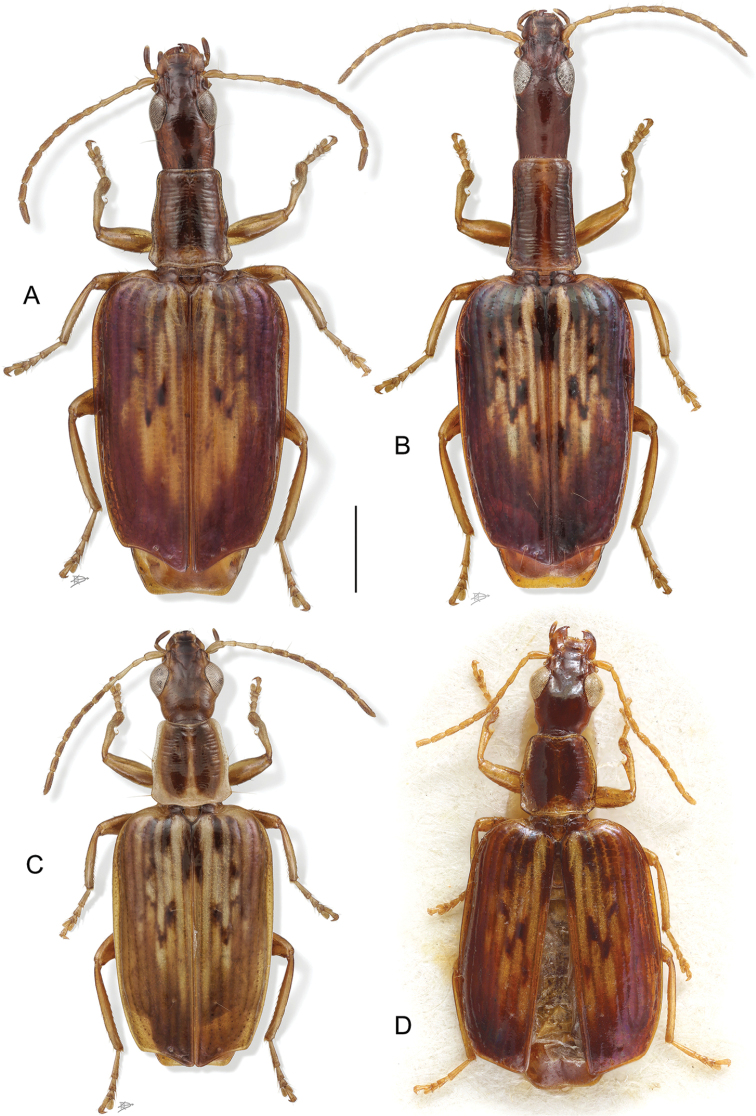
Example of Terry Erwin’s habitus illustrations: habitus of four *Straneotia* species. From Erwin and Aldebron (2018: fig. 14).

Excellent taxonomic publications clarify the morphological characters central to the identification of the given taxa. For this purpose, one often uses illustrations, usually drawings or photographs. Terry had his own style for such illustrations. His line drawings, which focus on the essential features and partly also emphasize them (see for an example Fig. 1), have been particularly helpful to me for identification of species. In his later works, photographs of the beetles’ habitus and other details are added (see for example Figs 2, 3). These effective photographs were mostly taken by professional photographers working in Smithsonian Museum’s Entomology Department, but Terry’s careful supervision ensured that they revealed the critical features required for accurate identifications.

His identification keys are generally suitable for taxonomic laypersons (e.g., Erwin and Kavanaugh 2000). This approach to taxonomy, not just for its own sake, but also as a service for other scientists, distinguishes great taxonomists like Terry from those with more limited influence, in my opinion. This personal carriage is amplified by Terry’s commitment to updating the last great work that Carl Lindroth was unfortunately unable to complete before his death. Terry revised of Lindroth’s key down to the generic level and wrote a chapter on a modern systematics of the northern carabids (Lindroth et al. 1985, 1986). In this way, Terry contributed to this work which is widely used by carabidologists, not solely from Scandinavia, but also from other European countries.

Working with a huge collection of beetles means that a curator must also engage with other collections, curators, and collectors. A broad appreciation of systematic work is required that extends into the historical dimension. In this context, Terry produced some fine papers that honored outstanding systematists like Maximillien de Chaudoir as well as citizen scientists like Max Bänninger (Erwin and Halpern 1978; Ball and Erwin 1983; Erwin 1983).

I was grateful to find a really extraordinary and useful work by Terry during a frustrating hour-long search for types in the dark, seemingly endless “canyons” between single collections of carabids in the Muséum national d’Histoire naturelle, Paris. He had created a presentation with maps to locate the specific carabidological collections stored in this huge museum and he made it available as a copy to the visitors. After I found this guide produced in 1971, it allowed me to efficiently succeed in meeting my goals 46 years later in 2017. This is a work of service that has lasting value! It has provided invaluable service to many carabidologists, I am sure of it! Any serious carabidologist who spends time in this fantastic museum will greatly appreciate this guide.

Joint awareness of an impediment to taxonomic publication and the central role of identification keys in organismic biology led Terry to initiate, with his friend, Lyubomir Penev, the taxonomic journal “ZooKeys” (Penev in Spence et al. 2021). Through Terry’s leadership, Pensoft launched and established “ZooKeys” as a well-respected international, peer-reviewed journal (Penev et al. 2008; Erwin et al. 2015) emphasizing rapid and effective taxonomic publication through modern web-based services and free access for readers (Erwin et al. 2012; Penev et al. 2012; Stoev et al. 2013). Clearly, “ZooKeys” has been a major accomplishment that supports modern work in zoological taxonomy as a basis for other biological disciplines.

## Natural history-based science

As most taxonomists are also interested in other facets of their favorite organisms, Terry was most interested in the habits, food, behavior, and ecology of ground beetles. Thus, the natural history of ground beetles became an important focus of his research. Natural history can be understood as a field of endeavor that deals with species in their natural environment and that leans more towards observational and descriptive studies than toward experiments.

Terry worked on numerous fascinating phenomena ranging from ectoparasitic lifestyles (Erwin 1967; Frank et al. 2009) and myrmecophily (Erwin 2013; Erwin and Amundson 2013) to the diving behavior of *Cicindis horni* Bruch, 1908 associated with eating fairy shrimp (Erwin and Aschero 2004). His work on larval development of *Brachinus* species in North America is particularly well known (Erwin 1966, 1967, 1972) and this stimulated Europeans to find out more about the natural history of their bombardier beetle species (e.g., Saska and Honek 2004).

Many of Terry’s observations have stimulated other entomologists and citizen scientists to observe and study behavior, development, and other aspects of the natural history of ground beetles. An example is his publication about the ectoparasitoidism of *Eurycoleus macularis* (Chevrolat, 1835) which develops on fungus beetles of the genus *Amphix* (Endomychidae), with larvae and adults both relying on the host as prey and living together with them (Erwin and Erwin 1976). More than 40 years later, Hunting and Yang (2019) reported that they found another percaline ground beetle in Taiwan, namely *Lioptera erotyloides* Bates, 1883, which is quite similar in coloration to fungus beetles of the subfamily Megalodacninae (Erotylidae). Ground beetles and fungus beetles were encountered together. However, the authors could not detect any trophic interaction between the beetles. *Lioptera erotyloides* is more widely distributed in subtropical East Asia and is also found in the Gutianshan National Park (China). Zumstein et al. (2021) were able to detect both this pericaline species and comparably colored erotyloids in flight interception traps there, but we were not able to observe the species alive in the field. However, the parallels shown here between ground beetles and fungus beetles are striking. Thus, Erwin and Erwin (1976) may have described a pattern of ectoparasitoid behavior for pericalines that is more widespread than previously known.

Erwin and Stork (1985) revised the Hiletini, a pantropically distributed group of ground beetles with large, hemispherical mandibles. Terry was fascinated by Hiletini species and spent many nights in the field watching them, hoping to discover more about their way of life. Although he published the only note about feeding habits of a member of this ground beetle tribe (Erwin 2011), I expect that he regretted being able to clearly understand how these beetles hunt and consume their prey using their distinctive mandibles.

Observational studies set bases for understanding trophic interactions, behavior, phenology, and many other phenomena. In a modern terminology natural history contributes to understanding ecological traits which are, in turn, essential for an understanding of the mechanisms which shape community structures and drive species to extinction (e.g., Gerisch et al. 2012; Homburg et al. 2014; Nolte et al. 2017). Terry’s natural history approach aimed to compile ecological traits of species across whole regions. His later regional monographs attempted to include all carabid species, and he aimed to develop comprehensive databases of ecological and distributional traits for this region. This approach resulted, for example, in the series ’A treatise on the Western Hemisphere Caraboidea (Coleoptera): Their classification, distribution, and ways of life’ (Erwin 2007, 2011; Erwin and Pearson 2008).

The importance of this kind of natural history is often underestimated, and its decline in academia is rather staggering (e.g., Tewksbury et al. 2014). However, there are now young scholars who emphasize the importance of natural history for a modern development of organismic biology (Christenhusz 2020). Thus, Terry’s work in natural history (and related ecological fields) doubtlessly contribute to essential basic work in organismic biology and we may hope help inspire a more emphasis on natural history as a basis for integration of systematics and ecology.

## Biodiversity studies and fogging

Terry’s publication about beetles collected by fogging on the tree species *Luehea seemannii* fired up a debate about arthropod species richness on Earth and was central to founding the discipline of biodiversity science (Erwin 1982). Although today most think that there are fewer species than Erwin first predicted based on his initial fogging study (e.g., Hamilton et al. 2013; Stork 2018), questions inspired by this work are still intensively discussed almost four decades later. These include, for example, relationships of species diversity among taxonomic groups (Garcia-Robledo et al. 2020), and the proper methods to upscale species numbers from small study sites to large areas (Basset et al. 2012; Schuldt et al. 2015; Staab and Schuldt 2020). Garcia-Robledo et al. (2020) further developed Terry’s arguments about the ratio between insect and plant species diversity into a diversity-ratio model, which is called by the authors the ‘Erwin Equation of Biodiversity’. In addition, these authors illustrate how Terry’s work inspired other scientists to refine biodiversity estimates to determine insect numbers more accurately.

In my opinion, the 1982 paper in “The Coleopterists Bulletin” is special not only in inspiring the diversity-ratio approach, but in the fact that Terry really tried measure species diversity in a defined area of tropical forest. He was among the first to bring this into the realm of science by framing general the effort to understand biodiversity in terms of hypothesis testing. Terry made it clear in the lively debate about his estimate of species number that the point was not whether he had been right or wrong, but that measurements should be used in science to test hypotheses. In this case the ultimate goal was providing the best estimate possible of the number of species on Earth based on measurements from the living world, rather than counts from taxonomic catalogues. He underscored this position in his contribution to the famous book edited by Edward O. Wilson, which ultimately initiated the introduction and thus acceptance of the term `biodiversity’ among scientists and the general public worldwide (Erwin 1988). Biodiversity emerged as a living concept instead of one scientifically reflected mainly in the non-living content of museums. The emphasis changed from counting up what was named and known, to trying to understand the dimensions of what was unknown about life on Earth.

Terry developed the fogging as a sampling method for his research about tropical canopy faunas. The general method had perhaps been first employed by Roberts (1973). However, Terry modified and standardized the fogging with insecticides and catapulted an appreciation of the technique into the general awareness of biologists worldwide (e.g., Erwin 1983). It is now mentioned in textbooks as an important sampling method (e.g., Samways et al. 2010; Gullan and Cranston 2014). Terry’s success in revealing the surprising arthropod abundance in canopies, including many new species, initiated many further studies of species diversity in this forest stratum that had long gone unnoticed (Stork 1991; Longino et al. 2002; Novotny and Basset 2005; Basset et al. 2012).

Terry not only used classical cladistic approaches in his research, but also explored the mechanisms leading to the enormous diversity of species, especially the life forms and radiations of carabids. He developed the ‘taxon pulse model’ to explain this extraordinary diversity (Erwin 1979, 1981, 1985). This hypothesis considered many aspects of ground beetle biology and proposed a unidirectional origin of arboreal carabids with their numerous morphological features from ground-dwelling ancestors. Overall, the hypothesis was innovative, comprehensible, and logical, and it was cited and discussed by numerous carabidologists, although modern molecular approaches have shown that carabids do not follow the postulated evolutionary paths as suspected (Ober 2003). Terry nonetheless appreciated that the idea had been tested scientifically.

## Conservation biology and ecology, especially of tropical rainforests

Terry was genuinely excited about ground beetles in tropical rainforests and spent as much time as possible over the past decades in the field. Thus, he experienced firsthand the destruction of rainforest and the threat to vast areas that remain. Conservation-related research therefore seemed important to him, but he did not forget his taxonomic roots. In fact, he wrote or co-authored several publications positioned at the interface between conservation biology and taxonomy (Erwin 1991; Kemmer et al. 1993; Kress et al. 1998; Erwin et al. 2005).

Terry’s extensive field experience, built up over decades, made him a highly desired cooperating partner in numerous projects. These projects addressed numerous questions of modern ecology and climate change that related directly to the biology of tropical rainforests. Examples include work about drought sensitivity (Phillips et al. 2009), forest biomass (Baker et al. 2004a, b), carbon cycle and storage (Fauset et al. 2015; Sullivan et al. 2017).

One of Terry’s contributions about conservation biology was published in Science (Erwin 1991) and prompted lively discussion (e.g., Moritz et al. 2000; Avise 2001; Brooks et al. 2006; Frankham et al. 2009). The paper has collected more than 200 citations through April 2021 according to the ISI Web of Science. In that paper, Terry explained the idea that areas with numerous young (and phylogenetically related) species are important for maintaining evolutionary processes that generate extraordinary species diversity. The tropical rainforest comprises many such areas and Terry reasoned that their evolutionary function must depend on a system of highly interconnected habitats to conserve the evolutionary and adaptive potential of radiating lineages on the long term (Erwin 1991). This, of course, makes a conceptual case for conservation of large tracts of rainforest habitat.

## Connectivity of Terry’s research activities

The many facets of Terry Erwin’s research activities are impressive in both depth and intellectual scope. However, most significant and likely most enduring aspects of Terry’s research relate to human understanding of the Carabidae and are best appreciated when the results are aggregated at the level of taxa.

**Figure 4.**
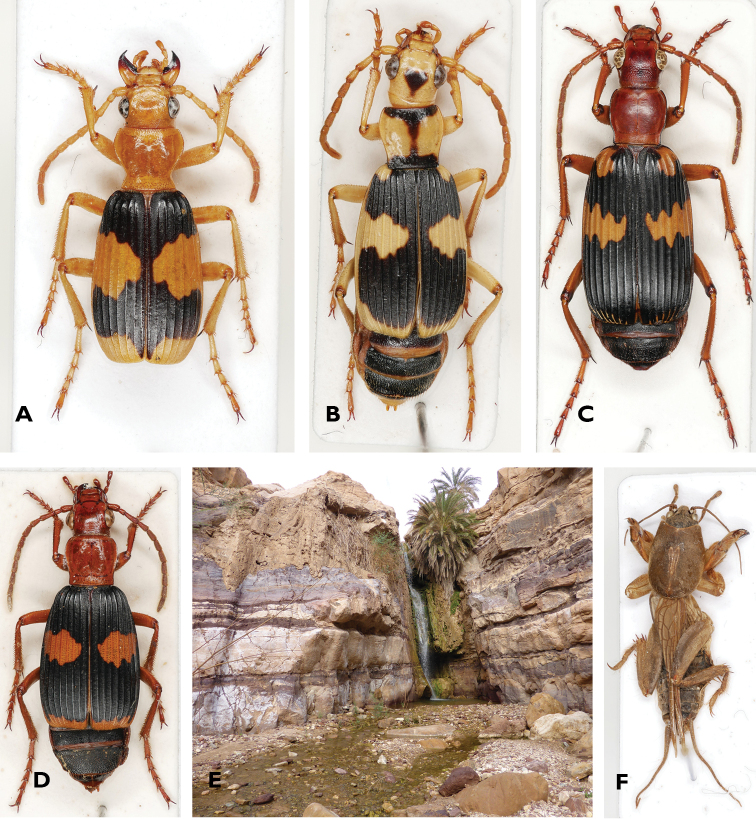
Bombardier beetles of the genus *Pheropsophus*, habitat of *Pheropsophus africanus*, and a *Gryllotalpa* specimen from the given habitat **A***P.* (s. str.) *aequinoctionalis* (Linnaeus, 1763) **B**P. (Stenaptinus) jessoensis A. Morawitz, 1862 **C**P. (Stenaptinus) hispanus (Dejean, 1824) **D**P. (Stenaptinus) africanus (Dejean, 1825) **E** habitat of *P. africanus* (Jordan, northeast of Dead Sea) **F***Gryllotalpa* spec.

The significance of this impact struck me in a wadi close to the Dead Sea, when students on a field trip found a large bombardier beetle of the genus *Pheropsophus* (Fig. 4). The defensive mechanism, incorporating explosive chemistry, heat-resistant enzymes, sound, and small quinone-containing puffs, was certainly impressive. Afterwards, the students learned that Terry Erwin had studied this group of ground beetles in North America and analyzed the hypermetamorphosis associated with the beetles’ most interesting ectoparasitic lifestyle. Given his taxon-based focus, Terry had worked on representatives of the genus *Pheropsophus* from both America and the eastern Palaearctic (Fig. 4A, B). According to his co-authored publication (Frank et al. 2009), the larvae of these species develop in egg chambers of mole crickets. Therefore, the students on my field trip started searching for mole crickets and their typical burrows close to the stream. We found *Gryllotalpa* species not only at all sites with *P. africanus* in North Africa and the Middle East (Fig. 4D, E, F), but also at those with *P. hispanus* in Andalusia and Morocco. Although we have not confirmed the intimate relationships between these brachinids and mole crickets, Terry’s work gave these students a place to begin unravelling trophic interactions between the western Palearctic *Pheropsophus* species (Fig. 4C, D) and gryllotalpids. The work of great biologists contributes in this way to a growing understanding of the Earth’s biota. In his life, Terry Erwin was an active and significant contributor to such scientific understanding and his work will continue to inspire students of natural history and the systematics of the Carabidae long after his passing.

For making so many fertile linkages for carabidology and broader scientific disciplines we must be deeply grateful to Terry. I am sure that he will be long remembered not only as a great person and gifted natural historian, but also as an outstanding, versatile, and productive scientist.
